# Phase 2a Pharmacokinetic, Safety, and Exploratory Efficacy Evaluation of Oral Gepotidacin (GSK2140944) in Female Participants with Uncomplicated Urinary Tract Infection (Acute Uncomplicated Cystitis)

**DOI:** 10.1128/AAC.00199-20

**Published:** 2020-06-23

**Authors:** J. Scott Overcash, Courtney A. Tiffany, Nicole E. Scangarella-Oman, Caroline R. Perry, Yu Tao, Mohammad Hossain, Aline Barth, Etienne F. Dumont

**Affiliations:** a eStudySite, La Mesa, California, USA; b GlaxoSmithKline, Collegeville, Pennsylvania, USA

**Keywords:** gepotidacin, uncomplicated urinary tract infection, acute uncomplicated cystitis, pharmacokinetics, safety

## Abstract

Gepotidacin, a triazaacenaphthylene bacterial type II topoisomerase inhibitor, is in development for treatment of uncomplicated urinary tract infection (uUTI). This phase 2a study in female participants with uUTI evaluated the pharmacokinetics (primary objective), safety, and exploratory efficacy of gepotidacin. Eligible participants (*n* = 22) were confined to the clinic at baseline, received oral gepotidacin at 1,500 mg twice daily for 5 days (on-therapy period; days 1 to 5), and returned to the clinic for test-of-cure (days 10 to 13) and follow-up (day 28 ± 3) visits. Pharmacokinetic, safety, clinical, and microbiological assessments were performed. Maximum plasma concentrations were observed approximately 1.5 to 2 h postdose. Steady state was attained by day 3. Urinary exposure over the dosing interval increased from 3,742 μg·h/ml (day 1) to 5,973 μg·h/ml (day 4), with trough concentrations of 322 to 352 μg/ml from day 3 onward. Gepotidacin had an acceptable safety-risk profile with no treatment-limiting adverse events and no clinically relevant safety trends. Clinical success was achieved in 19 (86%) and 18 (82%) of 22 participants at test-of-cure and follow-up visits, respectively. Eight participants had a qualifying baseline uropathogen (growth; ≥10^5^ CFU/ml). A therapeutic (combined clinical and microbiological [no growth; <10^3^ CFU/ml]) successful response was achieved in 6 (75%) and 5 (63%) of 8 participants at test-of-cure and follow-up visits, respectively. Plasma area under the free-drug concentration-time curve over 24 h at steady state divided by the MIC (*f*AUC_0–24_/MIC) and urine AUC_0–24_/MIC ranged from 6.99 to 90.5 and 1,292 to 121,698, respectively. Further evaluation of gepotidacin in uUTI is warranted. (This study has been registered in ClinicalTrials.gov under identifier NCT03568942.)

## Abstract (precis)

Gepotidacin, a triazaacenaphthylene bacterial type II topoisomerase inhibitor, is in development for treatment of uncomplicated urinary tract infection (uUTI). This phase 2a study in female participants with uUTI evaluated the pharmacokinetics (primary objective), safety, and exploratory efficacy of gepotidacin. Eligible participants (*n* = 22) were confined to the clinic at baseline, received oral gepotidacin at 1,500 mg twice daily for 5 days (on-therapy period; days 1 to 5), and returned to the clinic for test-of-cure (days 10 to 13) and follow-up (day 28 ± 3) visits.

## INTRODUCTION

Predominant uropathogens in uncomplicated urinary tract infections (uUTIs; acute uncomplicated cystitis) are Escherichia coli (75% to 90%), Staphylococcus saprophyticus (5% to 15%), and *Klebsiella*, *Enterobacter*, *Proteus*, and enterococcus uropathogens (5% to 10%) (1–3). Multidrug-resistant (MDR) uropathogens, commonly associated with nosocomial infections, have emerged at the community level, and treatment for uUTIs has become more difficult (4–6). Health authorities recognize extended-spectrum β-lactamase (ESBL)-producing *Enterobacteriaceae* as a serious threat (7) and drug-resistant *Enterobacteriaceae* as a critical priority pathogen (8). The MDR E. coli sequence type 131 clone has emerged as a cause of urinary tract infections and bacteremia worldwide (9–11). The availability of oral antimicrobials effective against ESBLs is limited and, for some outpatient infections, no oral options remain.

Gepotidacin (GSK2140944) is a triazaacenaphthylene bacterial type II topoisomerase inhibitor with a novel mode of action and with *in vitro* activity against most target pathogens resistant to established antibacterials (12–15). Phase 2 studies have demonstrated the efficacy of gepotidacin in acute bacterial skin and skin structure infections and uncomplicated urogenital gonorrhea (16–18). The microbiological activity of gepotidacin includes E. coli, the key causative uropathogen of uUTI, and S. saprophyticus, and Enterococcus faecalis. In addition, the efficacy of gepotidacin against E. coli was evaluated in a rat pyelonephritis model, which indicated potential efficacy in uUTI and supported clinical dose selection (19). The pharmacokinetics (PK) of gepotidacin have been well defined in healthy participants and demonstrated urine exposures that may support uUTI treatment (20–23). A phase 2a evaluation of oral gepotidacin in female participants with uUTI was conducted with the main objectives of evaluating plasma and urinary gepotidacin exposures and safety in this population. In addition, exploratory efficacy and PK/pharmacodynamic (PD) endpoints were assessed.

## RESULTS

### Participant disposition.

**FIG 1:**
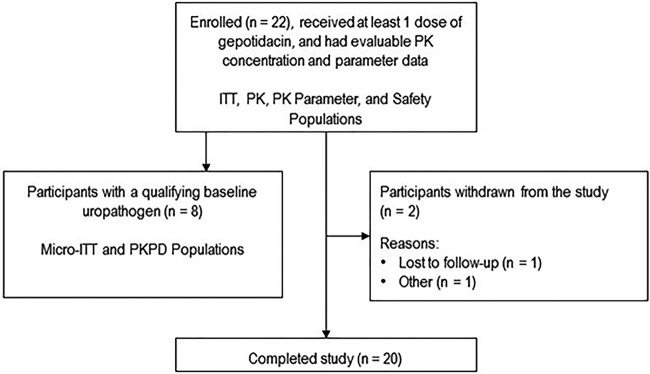
Participant disposition. ITT, intent-to-treat; micro, microbiological; PD, pharmacodynamic; PK, pharmacokinetic.

A total of 22 female participants with uUTI were enrolled and evaluated for PK, safety, and clinical efficacy in this phase 2a, single-center, single-arm, open-label study in the Unites States from July 2018 to January 2019 (see Fig. S1 in the supplemental material). Participants were confined to the clinic from baseline (days –1 to 1 predose) through the on-therapy (days 1 to 5) period and returned as outpatients for test-of-cure (TOC; days 10 to 13) and follow-up (day 28 ± 3) visits. Participants received oral gepotidacin at 1,500 mg twice daily (BID) for 5 days. Two participants (9%) withdrew from the study due to loss to follow-up and family reasons; there were no discontinuations due to adverse events (AEs) (Fig. 1).

### Participant baseline characteristics.

The majority of participants were White, ranged in age from 19 to 60 years, and had a body mass index ranging from 20.9 to 37.9 kg/m^2^ (Table 1). The number of past uUTI episodes ranged from 0 to 10 over the past 12 months across participants; the majority of participants reported ≤2 episodes.

**TABLE 1:** Baseline demographics (ITT population)

Demographic parameter^*a*^	Value for the parameter (*n* = 22)
Age (yr)	37.1 (12.26)
Reproductive status (no. [%])	
Postmenopausal	3 (14)
Sterile (of childbearing age)	1 (5)
Potentially able to bear children	18 (82)
Body mass index (kg/m^2^)	26.96 (5.366)
Height (cm)	163.22 (6.033)
Weight (kg)	72.01 (16.015)
Ethnicity (no. [%])	
Hispanic or Latino	6 (27)
Not Hispanic or Latino	16 (73)
Race (no. [%])	
Black or African American	4 (18)
White (White/Caucasian/European heritage)	18 (82)

a Unless otherwise indicated, values are means (standard deviations).

The mean total clinical symptom score at baseline was 7.9 (range, 4 to 12). All participants reported frequency and urgency, and all but 1 participant (5%) reported dysuria.

In the 22 intent-to-treat (ITT) participants, 19 baseline uropathogens were recovered, and 8 uropathogens from 8 participants (36%) met the qualifying uropathogen definition and inclusion in the microbiological intent-to-treat (micro-ITT) population (Fig. 1). This subset of 8 participants underwent both clinical and microbiological efficacy assessments.

### Pharmacokinetics.

**FIG 2:**
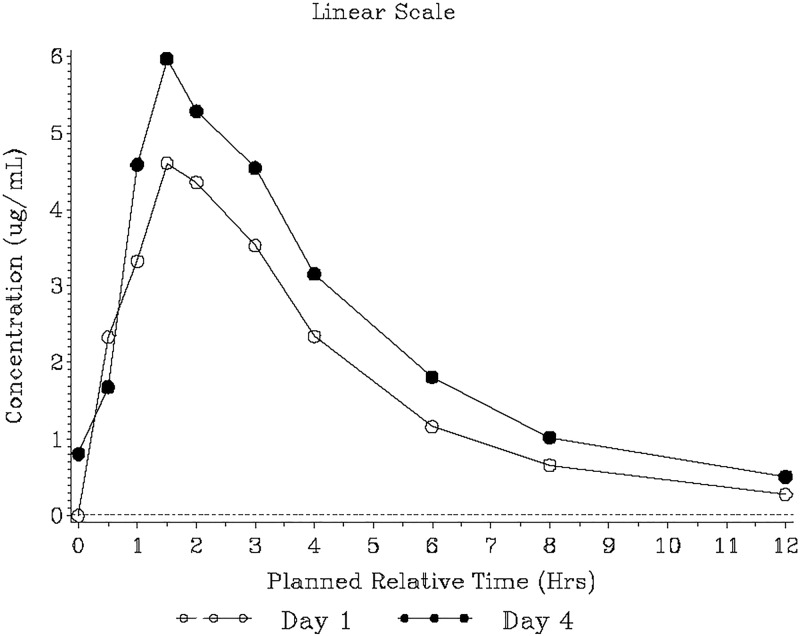
Median gepotidacin plasma concentration-time plot (pharmacokinetic population). The lower limit of quantification, represented by the dashed line, was 0.10 μg/ml. Day 1 plasma pharmacokinetic data after the 0.5-h collection for two participants were excluded due to vomiting. The 12-h pharmacokinetic data for one participant on day 1 and one participant on day 4 were excluded because the samples were collected after the second daily dose.

Median gepotidacin plasma concentrations peaked rapidly, with median maximum concentrations (*T*_max_) observed at 1.50 and 1.92 h postdose on days 1 and 4, respectively (Fig. 2). Concentrations declined in a multiphasic manner. Plasma exposure (maximum observed concentration [*C*_max_] and area under the concentration-time curve from time zero to the 12-h dosing interval [AUC_0–τ_]) was approximately 1.4-fold higher on day 4 than on day 1 (Table 2). The accumulation was consistent with an effective elimination half-life of 6.6 h. The between-participant variability in plasma exposures was moderate, with a higher coefficient-of-variation range for *C*_max_ (38% to 47%) than for the AUC_0–τ_ (29% to 32%) across days 1 and 4. Based on observed plasma predose concentrations (*C*_τ_) and statistical analysis, steady state was achieved by day 3 (Fig. S2 and Table S1).

**TABLE 2:** Summary of gepotidacin plasma PK parameters (PK parameter population) (*N* = 22)^*a*^

PK parameter and time point^*b*^	Value for the parameter^*c*^	
	Geometric mean (% CVb)	Min–max
*C*_max_ (μg/ml)		
Day 1	5.89 (47.3)	1.82–12.8
Day 2		
Day 3		
Day 4	8.44 (38.0)	3.82–16.8
Day 5		
*T*_max_ (h)		
Day 1	1.50^*d*^	0.470–3.07
Day 2		
Day 3		
Day 4	1.92^*d*^	0.450–4.12
Day 5		
AUC_0-τ_ (μg·h/ml)		
Day 1	20.2 (28.6)	11.0–31.0
Day 2		
Day 3		
Day 4	29.3 (31.8)	15.2–49.5
Day 5		
CL_ss_/*F* (l/h)		
Day 1		
Day 2		
Day 3		
Day 4	51.2 (31.8)	30.3–98.7
Day 5		
*R_o_*		
Day 1		
Day 2		
Day 3		
Day 4	1.40 (20.4)	1.09–2.20
Day 5		
*C*_τ_ (μg/ml)		
Day 1		
Day 2	0.621 (62.3)	0.122–1.84
Day 3	0.789 (37.4)	0.371–1.60
Day 4	0.851 (41.4)	0.460–1.99
Day 5	0.819 (46.4)	0.327–1.93

a *N*, number of participants in the treatment.

b *C*_max_, maximum observed concentration; *T*_max_, time of occurrence of *C*_max_; AUC_0-τ_, area under the concentration-time curve from time zero to the 12-h dosing interval; CL_ss_/*F*, apparent steady-state clearance; *R_o_*, accumulation ratio based on the AUC_0-τ_; *C*_τ_, predose concentration. The numbers of participants with evaluable PK parameter data were 20 for day 1 and 21 for days 2 to 5, with the exception that day 4 *R_o_* data are for 19 participants.

c CVb, between-participant geometric coefficient of variation; max, maximum; min, minimum.

d Values are medians.

**FIG 3:**
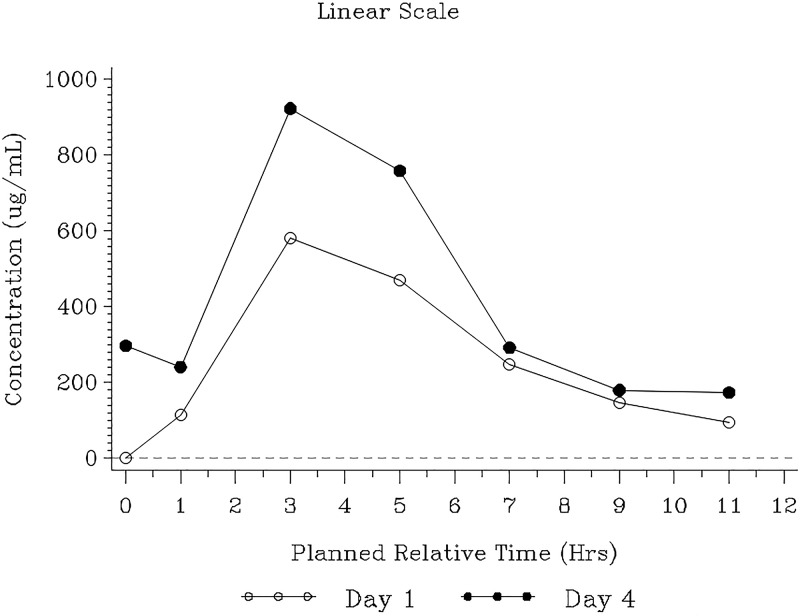
Median gepotidacin urine concentration-time plot (pharmacokinetic population). The lower limit of quantification, represented by the dashed line, was 1.00 μg/ml. Data are plotted by the planned relative midpoint time for each interval.

Median urine gepotidacin concentrations were generally higher on day 4 than on day 1 (Fig. 3). On day 1, approximately 20% of the dose was excreted in urine over the dosing interval, increasing to 31% on day 4 (Table 3). Overall exposure in urine (AUC_0–τ_) also increased from day 1 to day 4, with urine *C*_τ_ values ranging from 322 to 352 μg/ml from day 3 onward. The renal clearance values were similar on days 1 and 4. Approximately 460 mg of unchanged gepotidacin was excreted in urine over the steady-state dosing interval, with a minimum steady-state AUC_0–τ_ of 2,256 μg·h/ml.

**TABLE 3:** Summary of gepotidacin urine PK parameters (PK parameter population) (*N* = 22)^*a*^

PK parameter and time point^*b*^	Value for the parameter^*c*^	
	Geometric mean (% CVb)	Min–max
Ae_12_ (mg)		
Day 1	299 (107.6)	9.55–578
Day 2		
Day 3		
Day 4	460 (55.8)	135–1,100
Day 5		
AUC_0-τ_ (μg·h/ml)		
Day 1	3,742 (93.9)^*d*^	1,034–24,858
Day 2		
Day 3		
Day 4	5,973 (87.2)^*e*^	2,256–30,425
Day 5		
AUC_0–24_ (μg·h/ml)		
Day 1		
Day 2		
Day 3		
Day 4	11,945 (87.2)^*e*^	4,512–60,849
Day 5		
fe% (%)		
Day 1	19.9 (107.6)	0.637–38.5
Day 2		
Day 3		
Day 4	30.7 (55.8)	9.03–73.5
Day 5		
CLr (l/h)		
Day 1	14.8 (118.2)	0.420–41.5
Day 2		
Day 3		
Day 4	15.7 (45.2)	8.91–41.6
Day 5		
C_τ_ (μg/ml)		
Day 1		
Day 2	279 (154.7)	26.8–1,800
Day 3	322 (138.8)	42.1–3,670
Day 4	327 (248.7)	32.8–4,540
Day 5	352 (146.5)	68.2–4,010

a *N*, number of participants in the treatment.

b Ae_12_, total unchanged drug excreted over 12 h; AUC_0-τ_, area under the concentration-time curve from time zero to the 12-h dosing interval; AUC_0–24_, area under the concentration-time curve from 0 to 24 h; fe%, percentage of the given dose of drug excreted in urine; CLr, renal clearance; C_τ_, predose concentration. The numbers of participants with evaluable PK parameter data, except as otherwise noted, were 20 for days 1 and 2 and 21 for days 3 to 5. Day 1 urine PK parameter data for 2 participants were excluded from the summary statistics analysis due to vomiting.

c CVb, between-participant geometric coefficient of variation; max, maximum; min, minimum; *n*, number of participants with evaluable values.

d *n* = 16.

e *n* = 18.

Gepotidacin was measurable in cervical, rectal, and pharyngeal swabs on day 4, with the highest concentrations in rectal swabs (Table S2).

### Safety.

Twenty-one participants (95%) experienced AEs; gastrointestinal-related disorders had the highest prevalence (Table 4). Gastrointestinal AEs reported in >10% of participants were diarrhea, nausea, and vomiting and were the most prevalent drug-related events. Other drug-related AEs were vulvovaginal mycotic infection (2 participants, 9%), headache (1 participant, 5%), and chest discomfort (1 participant, 5%; noncardiac in nature).

**TABLE 4:** Summary of adverse events (safety population)

Event system organ class and preferred term	No. of events (%) (*n* = 22)
Any adverse event	21 (95)
Gastrointestinal disorders	21 (95)
Diarrhea	18 (82)
Nausea	17 (77)
Vomiting	5 (23)
Anal pruritus	1 (5)
Colitis	1 (5)
Dyspepsia	1 (5)
Eructation	1 (5)
Feces soft	1 (5)
Flatulence	1 (5)
Infections and infestations	6 (27)
Viral upper respiratory tract infection	2 (9)
Vulvovaginal mycotic infection	2 (9)
Gastroenteritis	1 (5)
Upper respiratory tract	1 (5)
Nervous system disorders	5 (23)
Headache	5 (23)
Musculoskeletal and connective tissue disorders	3 (14)
Back pain	2 (9)
Muscle spasms	1 (5)
Myalgia	1 (5)
General disorders and administration site conditions	2 (9)
Chest discomfort	2 (9)
Psychiatric disorders	1 (5)
Major depression	1 (5)
Respiratory, thoracic, and mediastinal disorders	1 (5)
Oropharyngeal pain	1 (5)

All AEs were mild (4 participants, 18%) or moderate (16 participants, 73%), except for a nonfatal serious AE of major depression with voluntary psychiatric hospitalization in 1 participant (5%) that occurred 9 days after the last dose and was considered by the investigator not to be related to gepotidacin. No participant experienced a drug-related AE of an intensity greater than moderate.

No clinically relevant laboratory changes were observed. Baseline and repeat urine dipstick results were consistent with the uUTI under study.

**FIG 4:**
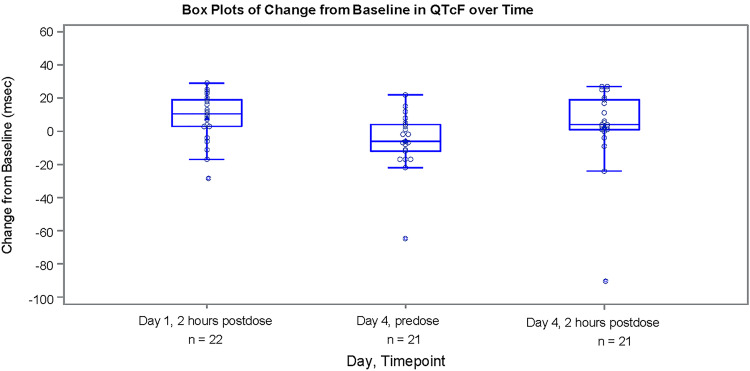
Box plot of change from baseline in QTcF over time (safety population). The triangle (inside the box) represents the mean value; the circle represents individual change from baseline; the top, middle, and bottom lines of the box represent the 75th, 50th (median), and 25th percentiles, respectively. The interquartile range is the distance between the 25th and 75th percentiles. The top and bottom whiskers represent maximum and minimum values, which are within 1.5× the interquartile range from the edge of the box, respectively. Any points outside the whiskers are deemed outliers. QTcF, QT interval corrected for heart rate according to Fridericia.

There were no clinically significant electrocardiogram (ECG) findings or changes from baseline. No participants had a QT interval corrected for heart rate according to Fridericia (QTcF) of ≥480 ms or an increase of >30 ms (Fig. 4). Mean QTcF (minimum, maximum) change from baseline to day 4 at 2 h postdose was 3.4 (–89, 27) ms. No clinically relevant changes in vital signs were observed.

### Exploratory efficacy.

**(i) Clinical.** In the ITT population, at TOC, clinical success was observed for 19 of 22 participants (86%; Clopper-Pearson 95% confidence interval [CI], 65% to 97%), and clinical failure was observed for 3 of 22 participants (14%; Clopper-Pearson 95% CI, 3% to 35%) (Table 5). Clinical success was achieved in 12 of 14 participants (86%) who did not have a qualifying baseline uropathogen, indicating complete resolution of clinical symptoms in these participants. Clinical response results were similar between TOC and follow-up visits (Table S3); however, per sponsor-determined clinical response, there was an additional clinical failure at follow-up.

**TABLE 5:** Summary of investigator-determined and sponsor-determined clinical outcomes and responses at test-of-cure by qualifying uropathogen isolated at baseline

Uropathogen group, clinical response (success or failure), and clinical outcome category	No. of participants by population and method (% [95% CI])^*a*^			
	Intent-to-treat population (*n* = 22)		Microbiological intent-to-treat population (*n* = 8)	
	Investigator-determined	Sponsor-determined	Investigator-determined	Sponsor-determined
All qualifying uropathogens (*n* = 8)				
Success	7 (88 [47 to >99])	7 (88 [47 to >99])	7 (88 [47 to >99])	7 (88 [47 to >99])
Clinical success	7 (88)	7 (88)	7 (88)	7 (88)
Failure	1 (13 [<1 to 53])	1 (13 [<1 to 53])	1 (13 [<1 to 53])	1 (13 [<1 to 53])
Clinical failure	1 (13)	1 (13)	1 (13)	1 (13)
Unable to determine	0	0	0	0
No qualifying uropathogen (*n* = 14)				
Success	12 (86 [57 to 98])	12 (86 [57 to 98])		
Clinical success	12 (86)	12 (86)		
Failure	2 (14 [2 to 43])	2 (14 [2 to 43])		
Clinical failure	0	0		
Unable to determine	2 (14)	2 (14)		
Total for groups (all participants)				
Success	19 (86 [65 to 97])	19 (86 [65 to 97])	7 (88 [47 to >99])	7 (88 [47 to >99])
Failure^*b*^	3 (14 [3 to 35])	3 (14 [3 to 35])	1 (13 [<1 to 53])	1 (13 [<1 to 53])

a A participant was counted more than once under a uropathogen category if multiple qualifying uropathogens within that uropathogen category were isolated at baseline for the participant. Other Gram-negative bacilli consisted of Citrobacter koseri (1) and Klebsiella pneumoniae (1). CI, confidence interval (Clopper-Pearson).

b No failures required an alternative antibiotic for treatment of uncomplicated urinary tract infection throughout the study.

**FIG 5:**
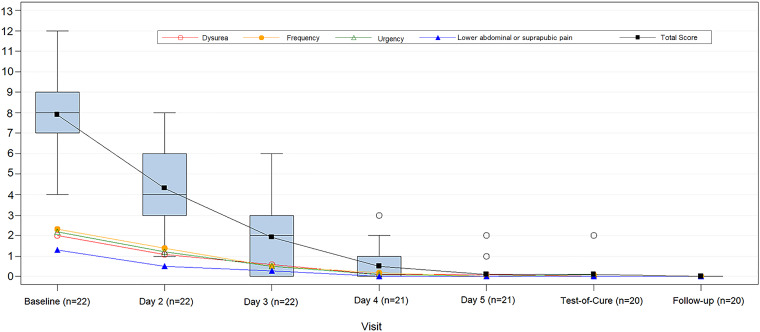
Individual clinical symptom score and box plot of total score over time (ITT population) (*n* = 22). The box represents the 25th to 75th percentiles. Within the box, the horizontal line represents the median, and the square indicates the mean. The upper and lower whiskers represent 1.5× the interquartile range. The open circles represent individual participant outlier scores.

The mean baseline total clinical symptom score (7.9) decreased to 0.1 and 0 at TOC and follow-up, respectively, with a similar trend for each symptom category (Fig. 5). All participants who completed the full course of gepotidacin and presented at TOC (*n* = 19) had complete symptom resolution; 1 participant who received only 6 doses of gepotidacin had a score of 2. All 20 participants with clinical scores reported at follow-up had a score of 0.

Clinical cure in most uropathogen groups was observed by approximately day 4 with an increase through TOC and follow-up.

**(ii) Microbiological.** The 8 qualifying baseline isolates consisted of 5 E. coli isolates and 1 isolate each of Citrobacter koseri, Klebsiella pneumoniae, and S. saprophyticus (Table 6). Of the 5 qualifying E. coli uropathogens, 2 were MDR, and 1 of those was both MDR and quinolone resistant.

**TABLE 6:** Uropathogens recovered at baseline (ITT population)

Group and uropathogen recovered^*a*^	No. (%) (*n* = 22)^*b*^
Total group	19
Acinetobacter pittii	1 (5)
Citrobacter freundii complex	1 (5)
Citrobacter koseri	1 (5)
Escherichia coli	14 (74)
MDR Escherichia coli	2 (11)
Quinolone-resistant Escherichia coli	1 (5)^*c*^
Klebsiella pneumoniae	1 (5)
Staphylococcus saprophyticus	1 (5)
Qualifying group (≥10^5^ CFU/ml)	8
Citrobacter koseri	1 (13)
Escherichia coli	5 (63)
MDR Escherichia coli	2 (25)
Quinolone-resistant Escherichia coli	1 (13)^*c*^
Klebsiella pneumoniae	1 (13)
Staphylococcus saprophyticus	1 (13)

a Multidrug resistant (MDR) refers to a uropathogen that was resistant to ≥3 relevant antibiotic classes.

b The denominator for the percentage calculations was the number of pathogens.

c Of the E. coli uropathogens, two were MDR and one of those was both MDR and quinolone resistant.

Most qualifying baseline uropathogens were resistant to ampicillin (5 of 8 isolates, 63%); none were resistant to nitrofurantoin, meropenem, or fosfomycin. No phenotypic ESBL-producing uropathogens were recovered. Against the 8 qualifying baseline uropathogens in the micro-ITT population, gepotidacin MIC values ranged from 0.06 to 4 μg/ml (Table S4).

For 7 of 8 participants (88%) in the micro-ITT population, no growth (eradication) was observed starting on day 2. At TOC, microbiological success was achieved in 7 of 8 participants (88%), including a participant with K. pneumoniae infection (Table 7 and Table S5). At TOC, there was 1 microbiological failure (13%) due to an indeterminant laboratory result (i.e., out-of-stability specimen); however, the participant was a clinical success. Microbiological response results were similar between TOC (Table S5) and follow-up (Table S6) visits except that there was an additional microbiological failure at follow-up. One participant had regrowth of C. koseri with no change in gepotidacin MIC or susceptibility.

**TABLE 7:** Summary of plasma and urine PK/PD, microbiological response, clinical response, and therapeutic response at TOC and follow-up by qualifying uropathogen isolated at baseline (micro-ITT population)^*a*^

Participant no.	Qualifying baseline uropathogen	Gepotidacin MIC (μg/ml)	Plasma *f*AUC_0-24_/MIC	Urine AUC_0–24_/MIC	Microbiological response		Clinical response		Therapeutic response	
					TOC	Follow-up	TOC	Follow-up	TOC	Follow-up
1	C. koseri	0.5	79.6	NA	S	F	S	S	S	F
2	E. coli	2	22.1	7,379	S	S	S	S	S	S
3	E. coli	0.5	90.5	121,698	S	S	S	S	S	S
4	E. coli	2	30.6	9,011	S	S	S	S	S	S
5	E. coli^*b*^	1	30.6	7,926	F^*c*^	F^*d*^	S	S	F	F
6	E. coli^*e*^	2	NA	NA	S	S	F^*f*^	F^*f*^	F	F
7	K. pneumoniae	4	6.99	1,292	S	S	S	S	S	S
8	S. saprophyticus	0.06	1,040	543,252	S	S	S	S	S	S

a AUC_0–24_, area under the concentration-time curve from time zero to 24 h; *f*AUC, area under the free-drug concentration-time curve; NA, not available (steady-state pharmacokinetic data not available); TOC, test-of-cure; S, success; F, failure.

b Isolate was multidrug resistant (e.g., resistant to ≥3 relevant antibiotic classes) and quinolone resistant.

c Microbiological failure due to an out-of-stability urine specimen.

d Microbiological failures at TOC were also considered microbiological failures at follow-up.

e Isolate was multidrug resistant.

f Participant received only 6 doses of gepotidacin due to withdrawal by the participant. The participant had a baseline total clinical score of 10 that decreased to 2 at TOC; however, that was not complete symptom resolution, and the clinical response was clinical failure. At follow-up, the total clinical symptom score was 0, which was a sponsor-determined clinical outcome of delayed clinical success; however, the clinical response remained a clinical failure per the analysis plan.

Growth was observed for 2 isolates posttreatment (1 E. coli isolate on day 3 and 1 C. koseri isolate at follow-up); both were resistant to ampicillin at baseline and the posttreatment time point. There was no change in the gepotidacin MIC (Table S4).

No participants had a baseline uropathogen that demonstrated a reduction in susceptibility to gepotidacin (i.e., ≥4-fold increase in gepotidacin MIC for baseline uropathogens versus postbaseline uropathogens of the same species and from the same participant) at any time point in the ITT population.

### (iii) Therapeutic response.

The overall participant-level therapeutic responses in the micro-ITT population were success for 6 of 8 participants (75%) and failure for 2 of 8 participants (25%) at TOC (Table 7 and Table S7). Details on clinical and microbiological failures leading to therapeutic response failures are described in the previous paragraphs (Table 7). Therapeutic response results were comparable between TOC and follow-up visits (Table 7 and Table S8); however, there was an additional microbiological failure at follow-up. No clinical or microbiological failures required an alternative antibiotic for treatment of uUTI throughout the study.

### Pharmacokinetic/pharmacodynamic.

Four out of the five participants with a qualifying E. coli uropathogen at baseline had evaluable PK/PD parameters (Table 7). Other qualifying baseline uropathogens were observed, but data were limited. Similar to the E. coli data, the PK parameter/MIC ratios were higher for the urine parameters than for the free-plasma parameters.

For the 4 participants with qualifying *Enterobacteriaceae* uropathogens who were also microbiological successes at TOC, the area under the free-drug concentration-time curve over 24 h at steady state divided by the plasma MIC (*f*AUC_0–24_/MIC) ranged from 6.99 to 90.5, and urine AUC_0–24_/MICs ranged from 1,292 to 121,698 (Table 7). The participant with the lowest plasma *f*AUC_0–24_/MIC (6.99) and urine AUC_0–24_/MIC (1,292) had a K. pneumoniae isolate with a gepotidacin MIC of 4 μg/ml and was a microbiological success.

## DISCUSSION

These results suggest that gepotidacin may provide a new oral treatment option for uUTI (acute uncomplicated cystitis) with further evaluation. The novel mechanism of action of gepotidacin could help meet the current need for oral antibacterial agents with activity against drug-resistant uropathogens (12–15).

The gepotidacin dose regimen in this population provided >600-fold-higher concentrations in urine than in free plasma at steady state, which is the target site of action for the treatment of uUTIs. The minimum gepotidacin urine concentrations remained above the gepotidacin MIC value of 4 μg/ml throughout the dosing interval. Of note, fluid intake was not standardized during the study; thus, any impact hydration status had on gepotidacin urinary exposures is unknown. The gepotidacin renal excretion for female participants with uUTI was higher than that of healthy participants with normal renal function or mild renal impairment (20% versus 7.5% of the dose), and day 1 plasma exposures in this study appeared to be higher than in previous phase 1 studies (mean *C*_max_ of 5.89 μg/ml versus 3.20 μg/ml) (unpublished data). Furthermore, gepotidacin concentrations were measurable in cervical, rectal, and pharyngeal swabs, supporting the evaluation of gepotidacin for gonorrhea (clinical trial NCT04010539).

An acceptable safety-risk profile was demonstrated after gepotidacin administration with no treatment-limiting AEs and no discontinuations due to AEs. The safety profile of gepotidacin was similar to that observed in previous studies. A high prevalence of gastrointestinal AEs was expected (16, 17, 20–23); however, the prevalence of gastrointestinal AEs in the current study (95%) was higher than that observed previously. This was the first study in all-female participants and in uUTI. The investigator observed that most nausea AEs had an acute onset within the first few doses and that tolerance was observed with repeat dosing.

Based on a previous gepotidacin corrected QT (QTc) evaluation (24), this study strategically included an on-treatment ECG assessment at the maximum steady-state gepotidacin exposures (i.e., day 4 at 2 h postdose); however, there were no cardiac AEs reported and no clinically significant ECG findings. In addition, there were no clinically relevant trends in the safety parameters.

In the ITT population (*n* = 22), symptom resolution (i.e., clinical score of 0) was achieved in 19 participants at TOC and in 20 participants at follow-up. The only participant without symptom resolution at TOC withdrew from study treatment and did not receive the full 5-day course of gepotidacin but underwent TOC and follow-up assessments. All participants had clinical efficacy observations consistent with expectations for a uUTI antibacterial.

Nonclinical models have shown that the PK/PD index most predictive of gepotidacin efficacy is *f*AUC/MIC (25). When the minimum exposure of gepotidacin in human urine at steady state measured in this study (minimum urine AUC_0–τ_ = 2,256 μg·h/ml; thus, minimum AUC_0–24_ = 4,512 μg·h/ml) and a gepotidacin MIC value of 4 μg/ml are applied, the minimum human urine AUC_0–24_/MIC achieved for the oral gepotidacin 1,500-mg BID dose exceeds the *f*AUC/MIC resistance suppression target of 275, as determined from an *in vitro* PK/PD hollow-fiber infection model (26), by approximately 4-fold, and 100% target attainment is expected in participants infected with uropathogens with gepotidacin MICs of ≤4 μg/ml.

This was a single-center evaluation in the United States, which led to very few drug-resistant isolates for evaluation. There was also a low prevalence of baseline uropathogens meeting growth criteria for the micro-ITT population (i.e., small sample size for microbiological assessment). The study was open label and did not include a comparator antibacterial. Global, multicenter, noninferiority gepotidacin phase 3 studies (clinical trials NCT04020341 and NCT04187144) should address these limitations.

The gepotidacin PK parameters were well defined in this female uUTI population. Oral gepotidacin at 1,500 mg BID for 5 days demonstrated an acceptable safety-risk profile (i.e., no discontinuations due to AEs) and provided positive exploratory efficacy findings, with no resistance development. These data support additional evaluation of gepotidacin in uUTI.

## MATERIALS AND METHODS

### Study population.

The study recruited nonpregnant females who were ≥18 and ≤65 years of age. Participants were required to have two or more of the following clinical signs and symptoms with onset ≤72 h at screening: dysuria, frequency, urgency, or lower abdominal pain. In addition, they were required to have pyuria (≥10 white blood cells/mm^3^ or the presence of leukocyte esterase) and/or nitrite from a pretreatment urine sample. Participants who had any preexisting condition that may have impacted gepotidacin absorption, distribution, metabolism, or excretion were excluded. Full inclusion and exclusion criteria are provided in the supplemental material.

This study was conducted in accordance with the Declaration of Helsinki and Good Clinical Practice guidelines. Protocol and procedures were reviewed and approved by an institutional review board. Written informed consent was obtained from participants before any study procedures were performed.

### Study design.

This was a phase 2a, single-center, single-arm, open-label study. Participants were confined to the clinic from baseline (days –1 to 1 predose) through the on-therapy period (days 1 to 5). Participants returned for outpatient visits at TOC (days 10 to 13) and follow-up (day 28 ± 3). Gepotidacin (1,500 mg; two 750-mg tablets) was administered orally BID for 5 days under site supervision. The target sample size was approximately 20 participants based on PK requirements.

### Pharmacokinetic assessments.

Serial blood and urine samples were collected from predose to 12 h postdose on day 1 (first dose) and day 4 (time matched to the first dose on day 1) (collection time points are given in Fig. S1 in the supplemental material). For steady-state assessment, predose blood (single collection) and urine (0- to 2-h interval) samples were collected before each time-matched dose on days 1 through 5.

For each plasma PK sample, 3 ml of whole blood was collected into tubes containing EDTA anticoagulant via an indwelling catheter and/or direct venipuncture. Each tube was inverted approximately 5 to 10 times immediately after the sample was drawn. The whole-blood sample may have been stored at room temperature for up to 60 min prior to centrifugation. The sample was centrifuged under refrigerated conditions (2°C to 8°C) at approximately 650 to 1,450 × *g*. Approximately 1.5 ml of plasma was transferred via a pipette into a 2-ml cryovial tube and kept frozen until analysis. Batched samples were shipped on dry ice to the bioanalytical laboratory for validated analysis.

For predose urine PK samples, a urine cup was used for collection. For all postdose urine PK samples, a urine jug was used for each collection interval. For each interval, approximately 1 ml of urine was transferred via a pipette into a 2-ml cryovial tube and kept frozen until analysis. Batched samples were shipped on dry ice to the bioanalytical laboratory for validated analysis.

Exploratory PK assessment included the collection of cervical, rectal, and pharyngeal swab specimens on day 4 (predose and 2 h postdose).

All PK samples were analyzed using validated ultra- or high-performance liquid chromatography with tandem mass spectroscopy methods by PPD Bioanalytical Laboratory (Middleton, WI).

### Safety assessments.

Adverse event monitoring, vital sign measurements, clinical laboratory evaluations, and ECGs, including on-treatment ECGs on days 1 and 4 matched with the 2-h PK collection, were performed.

### Exploratory efficacy assessments.

**(i) Clinical.** Clinical signs and symptoms of uUTI were recorded based on participant interview at baseline (pretreatment), days 2 through 5, TOC, and follow-up using a 0- to 3-point scale (0, none; 1, mild; 2, moderate; 3, severe) for the categories of dysuria, frequency, urgency, and lower abdominal or suprapubic pain (Fig. S3). At each on-therapy assessment, clinical success included both resolution of or improvement in signs and symptoms. Clinical success at TOC and follow-up was defined as resolution of signs and symptoms present at baseline (and no new signs and symptoms) and no use of other antimicrobial therapy for the current uUTI. At TOC, a score of zero was required for a participant to be deemed a clinical success. At follow-up, the participant must have had a score of zero at TOC that persisted from TOC to follow-up for a response of clinical success.

**(ii) Microbiological.** A urine sample was collected at baseline (pretreatment), predose days 2 through 5, TOC, and follow-up for Gram stain, quantitative bacteriology culture, and *in vitro* antimicrobial susceptibility testing using standard methods at a central laboratory (PPD Laboratories Central Lab, Highland Heights, KY). Susceptibility testing was conducted for all uropathogens by broth microdilution and gradient diffusion (fosfomycin only) according to guidelines of the Clinical and Laboratory Standards Institute (27, 28). Inclusion in the micro-ITT population required growth of a qualifying baseline uropathogen (≥10^5^ CFU/ml) (29, 30) (Fig. S4). Microbiological success was defined as culture-confirmed eradication (no growth; <10^3^ CFU/ml) of the qualifying baseline uropathogen. Multidrug resistance was defined as a baseline uropathogen that was resistant to ≥3 relevant antibiotic classes.

### Statistical analysis.

**(i) Analysis populations.** Analysis populations are defined in Table S9.

**(ii) Pharmacokinetic.** Noncompartmental PK analyses were performed using Phoenix WinNonlin, version 6.4 (Certara USA, Inc., Princeton, NJ), and SAS, version 9.3 (SAS Institute, Inc, Cary, NC), with actual sampling times. As total gepotidacin concentrations were measured in plasma, unbound values were derived by multiplying total concentrations by 0.67 to correct for the plasma protein binding of gepotidacin (33%) (unpublished data). Steady-state achievement was assessed using a linear mixed model with the Helmert transformation.

**(iii) Safety.** Adverse events, change from baseline values for clinical chemistry, hematology, vital signs, and ECG findings were summarized using SAS, version 9.3. Posthoc QTcF plots were generated using SAS, version 9.4.

**(iv) Exploratory efficacy.** Efficacy data were summarized by qualifying baseline uropathogen using counts and percentages, with the 95% Clopper-Pearson CI presented at TOC and follow-up using SAS, version 9.3.

Clinical outcome (investigator- and sponsor-determined) and response were summarized for the ITT and micro-ITT populations. Mean clinical symptom scores were summarized for the ITT population. Clinical cure was summarized for qualifying baseline uropathogens.

Microbiological outcome and response were summarized by predefined uropathogen groups or species. Urine quantitative bacteriology culture results were summarized. Results and interpretations of susceptibility testing for all uropathogens against gepotidacin and other antimicrobials were summarized.

Therapeutic response (success/failure), determined by statistical programming, was a measure of the overall efficacy response. Therapeutic success required both clinical success and microbiological success, or the participant was a deemed therapeutic failure. Therapeutic success was summarized by per-participant microbiological response and clinical response.

**(v) Pharmacokinetic/pharmacodynamic.** The plasma *f*AUC_0–24_/MIC and the urine AUC_0–24_/MIC ratio were determined using the day 4 PK parameters and the qualifying baseline uropathogen (day 1) MIC.

## Supplementary Material

Supplemental file 1
