# Supplementary material for: Phase 2a Pharmacokinetic, Safety, and Exploratory Efficacy Evaluation of Oral Gepotidacin (GSK2140944) in Female Participants with Uncomplicated Urinary Tract Infection (Acute Uncomplicated Cystitis)
Source: Antimicrob Agents Chemother. 2020 Jun 23;64(7):e00199-20. doi: 10.1128/AAC.00199-20 (PMC7318048; doi:10.1128/AAC.00199-20)
Supplement: Supplemental file 1 [file AAC.00199-20-s0001.pdf]

Supplementary Materials

Phase 2a Pharmacokinetic, Safety, and Exploratory Efficacy Evaluation of Oral Gepotidacin (GSK2140944) in Female Participants With Uncomplicated Urinary Tract Infection (Acute Cystitis)

J. Scott Overcash, Courtney A. Tiffany, Nicole E. Scangarella-Oman, Caroline R. Perry, Yu Tao, Mohammad Hossain, Aline Barth, Etienne F. Dumont

Supplementary contents:

Inclusion and Exclusion Criteria

FIG S1 Study design

FIG S2 Median gepotidacin predose trough plasma concentration-time plot on linear and semi-logarithmic scales (pharmacokinetic population)

FIG S3 Clinical signs and symptoms scoring for acute uncomplicated cystitis

FIG S4 Qualifying baseline algorithm for the micro-ITT population

TABLE S1 Statistical analysis of gepotidacin predose plasma concentrations (µg/ml) (pharmacokinetic parameter population)

TABLE S2 Summary of gepotidacin cervical, rectal, pharyngeal, and free plasma concentration-time data (µg/ml) (pharmacokinetic population)

TABLE S3 Summary of investigator-determined and sponsor-determined clinical outcome and response at follow-up by qualifying uropathogen isolated at baseline

TABLE S4 Distribution of gepotidacin MIC results by qualifying uropathogen (micro-ITT population)

TABLE S5 Summary of microbiological outcome and response at test-of-cure by qualifying uropathogen isolated at baseline (micro-ITT population)

TABLE S6 Summary of microbiological outcome and response at follow-up by qualifying uropathogen isolated at baseline (micro-ITT population)

TABLE S7 Summary of therapeutic response at test-of-cure (micro-ITT population)

TABLE S8 Summary of therapeutic response at follow-up (micro-ITT population)

TABLE S9 Analysis populations

Inclusion and Exclusion Criteria

Inclusion Criteria

Otherwise healthy participants were eligible to be included in the study only if all of the following criteria applied:

Age

1. Participant must have been ≥18 to ≤65 years of age, inclusive, at the time of signing the informed consent.

Type of Participant and Disease Characteristics

2. The participant had 2 or more of the following clinical signs and symptoms of acute cystitis with onset ≤72 hours of the screening assessment: dysuria, frequency, urgency, or lower abdominal pain.
3. The participant had pyuria (≥10 white blood cells/mm^3 or the presence of leukocyte esterase) and/or nitrite from a pretreatment clean-catch midstream urine sample based on local laboratory procedures.

   Note: Repeat baseline urine samples were allowed if contamination, defined as ≥10 squamous epithelial cells, was observed under microscopic evaluation.

Sex

4. The participant was female. A female participant was eligible to participate if she is not pregnant, not breastfeeding, and at least 1 of the following conditions applies:

   - Not a woman of childbearing potential (WOCBP)

   OR

   - A WOCBP who agrees to follow the contraceptive guidance from baseline through completion of test-of-cure (TOC).

Informed Consent

5. The participant was capable of giving signed informed consent, which included compliance with the requirements and restrictions listed in the informed consent form and in the protocol.

Exclusion Criteria

Participants were excluded from the study if any of the following criteria apply:

Medical Conditions

1. The participant resided in a nursing home or dependent care-type facility.
2. The participant had a body mass index ≥40.0 kg/m^2 or a body mass index ≥35.0 kg/m^2 with obesity-related health conditions such as high blood pressure or uncontrolled diabetes.

3. The participant had a history of sensitivity to the study treatment, or components thereof, or a history of a drug or other allergy that, in the opinion of the investigator or medical monitor, contraindicated her participation.
4. The participant was immunocompromised or had altered immune defenses that may have predisposed the participant to a higher risk of treatment failure and/or complications (e.g., renal transplant recipients, participants with clinically significant persistent granulocytopenia [absolute neutrophil count <1,000/μL], and participants receiving immunosuppressive therapy, including corticosteroid therapy [>40 mg/day prednisolone or equivalent for >1 week; ≥20 mg/day prednisolone or equivalent for >2 weeks, or prednisolone or equivalent ≥10 mg/day for >6 weeks]). Participants with a known CD4 count of <200 cells/mm^3 should not have been enrolled. Note: Participants with a positive test for human immunodeficiency virus were eligible for study participation.
5. The participant had uncontrolled diabetes, defined as a nonfasting glucose value >300 mg/dL or based on investigator judgment.
6. The participant had any of the following:
    - A medical condition that required medication that may have been aggravated by inhibition of acetylcholinesterase, such as:
        - Poorly controlled asthma or chronic obstructive pulmonary disease at baseline and, in the opinion of the investigator, not stable on current therapy
        - Acute severe pain, uncontrolled with conventional medical management
        - Active peptic ulcer disease
        - Parkinson disease
        - Myasthenia gravis
        - A history of seizure disorder requiring medications for control (this does not include a history of childhood febrile seizures)

    OR

    - Any surgical or medical condition (active or chronic) that may have interfered with drug absorption, distribution, metabolism, or excretion of the study drug (e.g., ileostomy or malabsorption syndrome). Participants who had a gastric bypass or a cholecystectomy were excluded from the study.

    OR

    - Hemoglobin value <12 g/dL or a known uncorrected iron deficiency.
7. The participant, in the judgment of the investigator, would not be able or willing to comply with the protocol or complete study follow-up.
8. The participant had a serious underlying disease that could have been imminently life threatening, or the participant was unlikely to survive for the duration of the study period.

Urinary Tract Infection/Renal/Urogenital Exclusions

9. The participant had acute cystitis that was known or suspected to be due to fungal, parasitic, or viral pathogens; or known or suspected to be due to *Pseudomonas aeruginosa* or *Enterobacteriaceae* (other than *Escherichia coli*) as the contributing pathogen.
10. The participant had symptoms known or suspected to be caused by another disease process such as asymptomatic bacteriuria or chronic interstitial cystitis.
11. The participant had an anatomical or physiological anomaly that predisposed the participant to urinary tract infections (UTIs) or may have been a source of persistent bacterial colonization, including calculi, obstruction or stricture of the urinary tract, primary renal disease (e.g., polycystic renal disease), or neurogenic bladder, or the participant had a history of anatomical or functional abnormalities of the urinary tract (e.g., chronic vesico-ureteral reflux, detrusor insufficiency).
12. The participant had an indwelling catheter, nephrostomy, ureter stent, or other foreign material in the urinary tract.
13. The participant who, in the opinion of the investigator, had an otherwise complicated UTI, an active upper UTI (e.g., pyelonephritis, urosepsis), signs and symptoms onset ≥96 hours before the screening assessment, or a temperature ≥101°F, flank pain, chills, or any other manifestations suggestive of upper UTI.
14. The participant had anuria, oliguria, or significant impairment of renal function (creatinine clearance <30 ml/min or clinically significant elevated serum creatinine).
15. The participant presented with vaginal discharge at baseline (e.g., suspected sexually transmitted disease).

Cardiac Exclusions

16. The participant had congenital long QT syndrome or known prolongation of the corrected QT (QTc) interval.
17. The participant had uncompensated heart failure, defined as New York Heart Association Class ≥III.
18. The participant had severe left ventricular hypertrophy.
19. The participant had a family history of QT prolongation or sudden death.
20. The participant had a recent history of vasovagal syncope or episodes of symptomatic bradycardia or bradyarrhythmia within the last 12 months.
21. The participant was taking QT-prolonging drugs or drugs known to increase the risk of torsades de points (TdP) per the www.crediblemeds.org “Known Risk of TdP” category at baseline, which could not be safely discontinued from baseline to TOC; or the participant was taking a strong cytochrome P450 enzyme 3A4 inhibitor or a strong P-glycoprotein inhibitor.

Cardiac Electrocardiogram Exclusion

22. The participant had a QTc >450 msec or a QTc >480 msec for participants with bundle-branch block. Note: The QTc was the QT interval corrected for heart rate according to either Bazett or Fridericia formula, machine, or manual overread.

Hepatic Exclusions

23. The participant had a known alanine transferase value >2 × upper limit of normal (ULN).
24. The participant had a known bilirubin value >1.5 × ULN (isolated bilirubin >1.5 × ULN was acceptable if bilirubin is fractionated and direct bilirubin <35%).
25. The participant had a current or chronic history of liver disease, or known hepatic or biliary abnormalities (with the exception of Gilbert's syndrome or asymptomatic gallstones), including symptomatic viral hepatitis or moderate-to-severe liver insufficiency (Child Pugh class B or C). Note: Participants with asymptomatic viral hepatitis were eligible for study participation.

Prior Antibiotic/Antifungal Use Exclusion

26. The participant received treatment with other systemic antimicrobials or systemic antifungals within 1 week before study entry.

Concomitant Medication Use Exclusion

27. The participant must have agreed not to use the medications or nondrug therapies from baseline through TOC (as described in the protocol).

Prior/Concurrent Clinical Study Experience

28. The participant was previously enrolled in this study or has previously been treated with gepotidacin.
29. The participant participated in a clinical trial and received an investigational product within 30 days or 5 half-lives, whichever is longer.

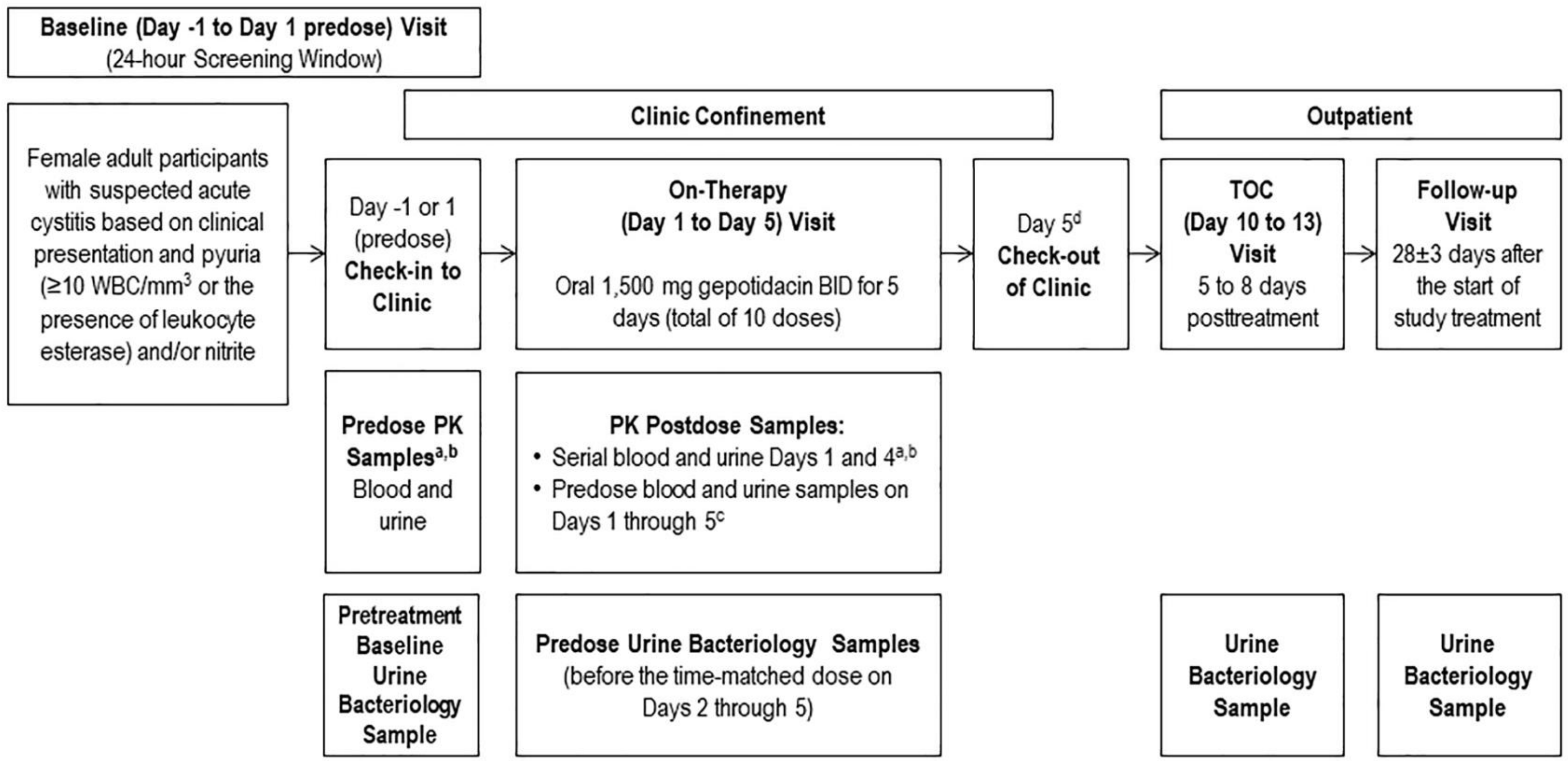

FIG S1 Study design.

[a] Serial blood PK sampling was performed for the first dose of gepotidacin on Day 1 and for the time-matched dose on Day 4. Blood samples were collected predose and at 0.5, 1, 1.5, 2, 3, 4, 6, 8, and 12 hours postdose. Optional cervical, rectal, and pharyngeal swab PK specimens were collected on Day 4 at predose and 2 hours postdose.

[b] Serial urine PK sampling was performed for the first dose of gepotidacin on Day 1 and for the time-matched dose on Day 4. Urine samples were collected predose and at intervals of 0 to 2 hours, 2 to 4 hours, 4 to 6 hours, 6 to 8 hours, 8 to 10 hours, and 10 to 12 hours postdose.

[c] Predose PK blood samples were collected before each time-matched dose on Days 1 through 5. Predose PK urine samples were collected 0 to 2 hours before each time-matched dose on Days 1 through 5.

[d] Participants checked-out of the clinic after all study procedures were performed, including a predose clean-catch midstream urine sample for Gram stain, quantitative bacteriology culture, and antimicrobial susceptibility testing, predose PK sample collections, and safety assessments. Participants should have remained in the clinic to complete a total of 10 doses. Participants were instructed to return for the TOC (Day 10 to 13) and follow-up (Day 28±3) Visits.

BID, twice daily; PK, pharmacokinetic; TOC, test-of-cure; WBC, white blood cells.

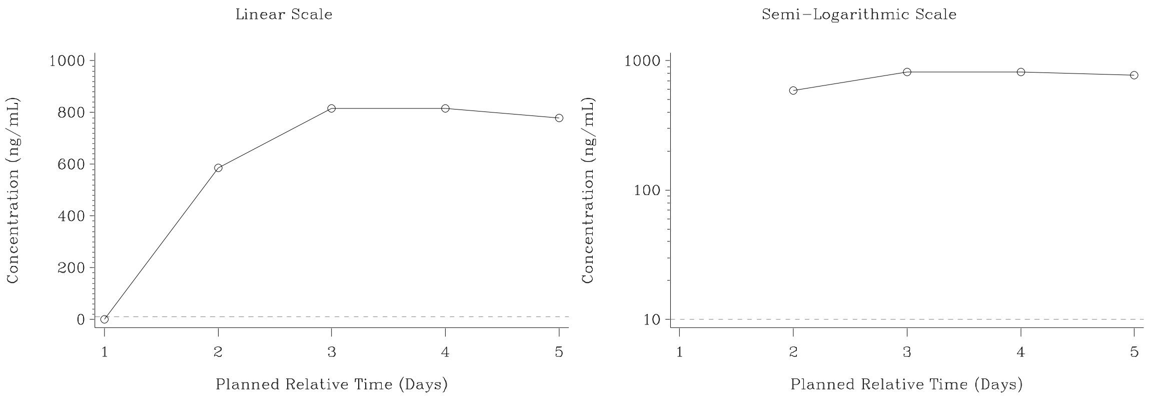

FIG S2 Median gepotidacin predose trough plasma concentration-time plot on linear and semi-logarithmic scales (PK population).

Concentrations are presented in ng/ml, whereas other pharmacokinetic data in the manuscript are presented as µg/ml. Lower limit of quantification (LLOQ) was 10.0 ng/ml. Dashed line represents LLOQ.

Acute cystitis clinical signs and symptoms will be scored as follows:

Clinical Signs and Symptoms	**None** Participants' normal clinical presentation prior to current infection	**Mild** Symptom is easily tolerated, causing minimal discomfort and not interfering with everyday activities	**Moderate** Symptom is sufficiently discomforting to interfere with normal everyday activities	**Severe** Symptom prevents normal everyday activities
	SCORE 0	SCORE 1	SCORE 2	SCORE 3
Dysuria				
Frequency				
Urgency				
Lower abdominal or suprapubic pain				

Scores will be recorded by the investigator or a qualified designee. When possible, the same scorer will be used at all assessment time points.

FIG S3 Clinical signs and symptoms scoring for acute uncomplicated cystitis

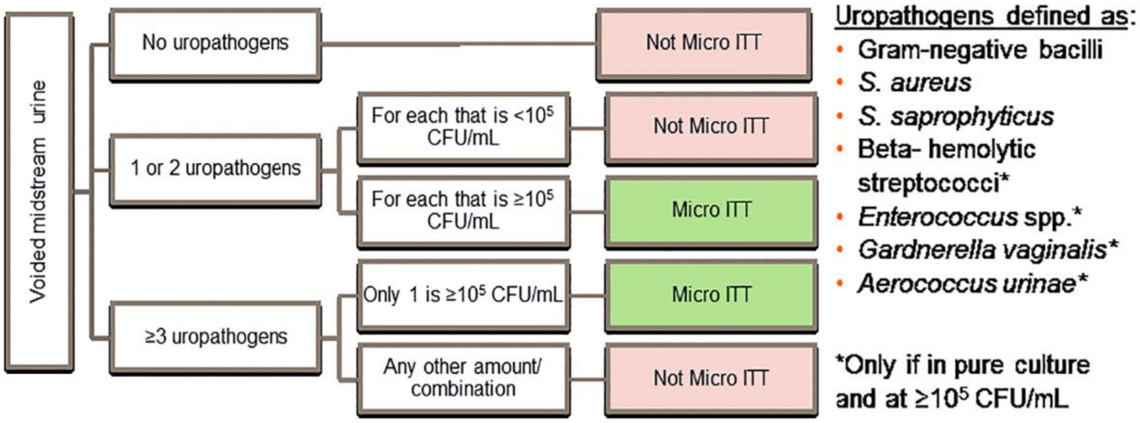

FIG S4 Qualifying baseline algorithm for the micro-ITT population.

TABLE S1 Statistical analysis of gepotidacin predose plasma concentrations (µg/ml) (pharmacokinetic parameter population)

Day	Predose geometric LS means	Comparison	Ratio of geometric LS means	95% CI	P-value
2	0.610	Day 2 versus Day 3 to Day 5	0.743	(0.640, 0.861)	0.0002
3	0.770	Day 3 versus Day 4 to Day 5	0.908	(0.776, 1.063)	0.2235
4	0.864	Day 4 versus Day 5	1.040	(0.869, 1.244)	0.6669
5	0.831	–	–	–	–

A linear mixed-model using day as fixed effect and participant as a random effect on the ln-transformed predose values (including the 12-hour postdose values on Day 1 and Day 4) was performed to evaluate if steady state was achieved using the Helmert transformation approach.
Abbreviations: CI, confidence interval; LS, least-squares.

TABLE S2 Summary of gepotidacin cervical, rectal, pharyngeal, and free plasma concentration-time data (µg/ml) (pharmacokinetic population)[a]

Matrix	N	Day	Planned relative time	n	Number imputed	Mean	%CV	Minimum	Maximum
Cervical	18	4	Predose	18	0	1.30	131.3	0.0601	5.94
		4	2 hours postdose	18	0	1.03	114.8	0.0190	4.25
Rectal	18	4	Predose	18	0	6.04	156.9	0.0678	36.30
		4	2 hours postdose	18	0	12.3	214.6	0.0989	90.90
Pharyngeal	20	4	Predose	19	0	0.0330	106.4	0.00275	0.165
		4	2 hours postdose	20	0	0.140	87.3	0.0118	0.527
Free plasma	21	4	Predose	21	0	0.618	44.2	0.308	1.33
		4	2 hours postdose	21	0	3.94	42.3	0.938	7.50

[a]%CV, coefficient of variation; n, number of participants with evaluable values; N, number of participants in the treatment.

TABLE S3 Summary of investigator-determined and sponsor-determined clinical outcome and response at follow-up by qualifying uropathogen isolated at baseline

**Qualifying uropathogen**<br>**Clinical response, n (%) (95% CI)[a]**<br>**Clinical outcome**	**Intent-to-Treat population N = 22**		**Microbiological Intent-to-Treat population N = 8**	
	**Investigator-Determined**	**Sponsor-Determined**	**Investigator-Determined**	**Sponsor-Determined**
All qualifying uropathogens				
N	8	8	8	8
Success	7 (88) (47 - >99)	7 (88) (47 - >99)	7 (88) (47 - >99)	7 (88) (47 - >99)
Sustained clinical success, n (%)	7 (88)	7 (88)	7 (88)	7 (88)
Failure	1 (13) (<1 - 53)	1 (13) (<1 - 53)	1 (13) (<1 - 53)	1 (13) (<1 - 53)
Delayed clinical success, n (%)	0	1 (13)	0	1 (13)
Clinical failure, n (%)	1 (13)	0	1 (13)	0
Clinical recurrence, n (%)	0	0	0	0
Unable to determine, n (%)	0	0	0	0
No qualifying uropathogen				
N	14	14	–	–
Success	12 (86) (57 - 98)	11 (79) (49 - 95)	–	–
Sustained clinical success, n (%)	12 (86)	11 (79)	–	–
Failure	2 (14) (2 - 43)	3 (21) (5 - 51)	–	–
Delayed clinical success, n (%)	0	1 (7)	–	–
Clinical failure, n (%)	0	0	–	–
Clinical recurrence, n (%)	0	0	–	–
Unable to determine, n (%)	2 (14)	2 (14)	–	–
All participants				
Success	19 (86) (65 - 97)	18 (82) (60 - 95)	7 (88) (47 - >99)	7 (88) (47 - >99)
Failure	3 (14) (3 - 35)	4 (18) (5 - 40)	1 (13) (<1 - 53)	1 (13) (<1 - 53)

A participant was counted more than once under a uropathogen category if multiple qualifying uropathogens within that uropathogen category were isolated at baseline for the participant. Other gram-negative bacilli consisted of *Citrobacter koseri* (1) and *Klebsiella pneumoniae* (1).
Abbreviations: CI, confidence interval.
[a]Clopper-Pearson CI.

TABLE S4 Distribution of gepotidacin MIC results by qualifying uropathogen (micro-ITT population)

**Qualifying uropathogen**<br>**Visit**	**N**	**Gepotidacin MIC (μg/ml)**<br>**≤0.03**	**0.06**	**0.12**	**0.25**	**0.5**	**1**	**2**	**4**	**8**
Escherichia coli										
Baseline	**5**	–	–	–	–	**1**	**1**	**3**	–	–
Day 3	**1**	–	–	–	–	–	–	**1**	–	–
Test-of-Cure	–	–	–	–	–	–	–	–	–	–
Follow-up	–	–	–	–	–	–	–	–	–	–
Other gram-negative bacilli[a]										
Baseline	**2**	–	–	–	–	**1**	–	–	**1**	–
Test-of-Cure	–	–	–	–	–	–	–	–	–	–
Follow-up	**1**	–	–	–	–	**1**	–	–	–	–
Staphylococcus saprophyticus										
Baseline	**1**	–	**1**	–	–	–	–	–	–	–
Test-of-Cure	–	–	–	–	–	–	–	–	–	–
Follow-up	–	–	–	–	–	–	–	–	–	–

[a]Other gram-negative bacilli consisted of *Citrobacter koseri* (1) and *Klebsiella pneumoniae* (1).

TABLE S5 Summary of microbiological outcome and response at test-of-cure by qualifying uropathogen isolated at baseline (micro-ITT population)

Qualifying uropathogen<br>Microbiological response, n (%) (95% CI)[a]<br>Microbiological outcome	Total<br>N = 8
Escherichia coli	
N	5
Microbiological success	4 (80) (28 - >99)
Microbiological eradication, n (%)	4 (80)
Microbiological failure	1 (20) (<1 - 72)
Microbiological persistence, n (%)	0
Microbiological recurrence, n (%)	0
Unable to determine, n (%)	1 (20)
Other gram-negative *b*acilli	
N	2
Microbiological success	2 (100) (16 - 100)
Microbiological eradication, n (%)	2 (100)
Microbiological failure	0 (0 - 84)
Microbiological persistence, n (%)	0
Microbiological recurrence, n (%)	0
Unable to determine, n (%)	0
Staphylococcus saprophyticus	
N	1
Microbiological success	1 (100) (3 - 100)
Microbiological eradication, n (%)	1 (100)
Microbiological failure	0 (0 - 98)
Microbiological persistence, n (%)	0
Microbiological recurrence, n (%)	0
Unable to determine, n (%)	0
All qualifying uropathogens	
N	8
Microbiological success	7 (88) (47 - >99)
Microbiological eradication, n (%)	7 (88)
Microbiological failure	1 (13) (<1 - 53)
Microbiological persistence, n (%)	0
Microbiological recurrence, n (%)	0
Unable to determine, n (%)	1 (13)
Participant level	
Microbiological success	7 (88) (47 - >99)
Microbiological failure	1 (13) (<1 - 53)

A participant was counted more than once under a uropathogen category if multiple qualifying uropathogens within that uropathogen category were isolated at baseline for the participant. A microbiological outcome of unable to determine was considered a microbiological failure. Other gram-negative bacilli consisted of *Citrobacter koseri* (1) and *Klebsiella pneumoniae* (1).
Abbreviations: CI, confidence interval.
[a]Clopper-Pearson CI.

TABLE S6 Summary of microbiological outcome and response at follow-up by qualifying uropathogen isolated at baseline (micro-ITT population)

**Qualifying uropathogen**<br>**Microbiological response, n (%) (95% CI)[a]**<br>**Microbiological outcome**	**Total**<br>**N = 8**
Escherichia coli	
N	5
Microbiological success	4 (80) (28 - >99)
Sustained microbiological eradication, n (%)	4 (80)
Microbiological failure	1 (20) (<1 - 72)
Sustained microbiological eradication[b], n (%)	1 (20)
Microbiological recurrence, n (%)	0
Unable to determine, n (%)	0
Other gram-negative *b*acilli	
N	2
Microbiological success	1 (50) (1 - 99)
Sustained microbiological eradication, n (%)	1 (50)
Microbiological failure	1 (50) (1 - 99)
Sustained microbiological eradication[b], n (%)	0
Microbiological recurrence, n (%)	1 (50)
Unable to determine, n (%)	0
Staphylococcus saprophyticus	
N	1
Microbiological success	1 (100) (3 - 100)
Sustained microbiological eradication, n (%)	1 (100)
Microbiological failure	0 (0 - 98)
Sustained microbiological eradication[b], n (%)	0
Microbiological recurrence, n (%)	0
Unable to determine, n (%)	0
All qualifying uropathogens	
N	8
Microbiological success	6 (75) (35 - 97)
Sustained microbiological eradication, n (%)	6 (75)
Microbiological failure	2 (25) (3 - 65)
Sustained microbiological eradication[b], n (%)	1 (13)
Microbiological recurrence, n (%)	1 (13)
Unable to determine, n (%)	0
Participant level	
Microbiological success	6 (75) (35 - 97)
Microbiological failure	2 (25) (3 - 65)

A participant was counted more than once under a uropathogen category if multiple qualifying uropathogens within that uropathogen category were isolated at baseline for the participant. Other gram-negative bacilli consisted of *Citrobacter koseri* (1) and *Klebsiella pneumoniae* (1).

Abbreviations: CI, confidence interval.

[a]Clopper-Pearson CI.

[b]Participants considered microbiological failures at test-of-cure were considered microbiological failures at follow-up. A microbiological outcome of unable to determine was considered a microbiological failure.

TABLE S7 Summary of therapeutic response at test-of-cure (micro-ITT population)

**Qualifying uropathogen**<br>**Therapeutic response, n (%) (95% CI)[a]**<br>**Microbiological per-participant response/clinical response**	**Total**<br>**N = 8**
Escherichia coli	
N	5
Success	3 (60) (15 - 95)
Microbiological success/clinical success, n (%)	3 (60)
Failure	2 (40) (5 - 85)
Microbiological success/clinical failure, n (%)	1 (20)
Microbiological failure/clinical success, n (%)	1 (20)
Microbiological failure/clinical failure, n (%)	0
**Other gram-negative bacilli**	
N	2
Success	2 (100) (16 - 100)
Microbiological success/clinical success, n (%)	2 (100)
Failure	0 (0 - 84)
Microbiological success/clinical failure, n (%)	0
Microbiological failure/clinical success, n (%)	0
Microbiological failure/clinical failure, n (%)	0
Staphylococcus saprophyticus	
N	1
Success	1 (100) (3 - 100)
Microbiological success/clinical success, n (%)	1 (100)
Failure	0 (0 - 98)
Microbiological success/clinical failure, n (%)	0
Microbiological failure/clinical success, n (%)	0
Microbiological failure/clinical failure, n (%)	0
**All qualifying uropathogens**	
N	8
Success	6 (75) (35 - 97)
Microbiological success/clinical success, n (%)	6 (75)
Failure	2 (25) (3 - 65)
Microbiological success/clinical failure, n (%)	1 (13)
Microbiological failure/clinical success, n (%)	1 (13)
Microbiological failure/clinical failure, n (%)	0
**Participant level**	
Success	6 (75) (35 - 97)
Failure	2 (25) (3 - 65)

A participant was counted more than once under a uropathogen category if multiple qualifying uropathogens within that uropathogen category were isolated at baseline for the participant. Other gram-negative bacilli consisted of *Citrobacter koseri* (1) and *Klebsiella pneumoniae* (1).

Abbreviations: CI, confidence interval.

[a]Clopper-Pearson CI.

TABLE S8 Summary of therapeutic response at follow-up (micro-ITT population)

**Qualifying uropathogen**<br>**Therapeutic response, n (%) (95% CI)[a]**<br>**Microbiological per-participant response/clinical response**	**Total**<br>**N = 8**
Escherichia coli	
N	5
Success	3 (60) (15 - 95)
Microbiological success/clinical success, n (%)	3 (60)
Failure	2 (40) (5 - 85)
Microbiological success/clinical failure, n (%)	1 (20)
Microbiological failure/clinical success, n (%)	1 (20)
Microbiological failure/clinical failure, n (%)	0
Other gram-negative bacilli	
N	2
Success	1 (50) (1 - 99)
Microbiological success/clinical success, n (%)	1 (50)
Failure	1 (50) (1 - 99)
Microbiological success/clinical failure, n (%)	0
Microbiological failure/clinical success, n (%)	1 (50)
Microbiological failure/clinical failure, n (%)	0
Staphylococcus saprophyticus	
N	1
Success	1 (100) (3 - 100)
Microbiological success/clinical success, n (%)	1 (100)
Failure	0 (0 - 98)
Microbiological success/clinical failure, n (%)	0
Microbiological failure/clinical success, n (%)	0
Microbiological failure/clinical failure, n (%)	0
All qualifying uropathogens	
N	8
Success	5 (63) (24 - 91)
Microbiological success/clinical success, n (%)	5 (63)
Failure	3 (38) (9 - 76)
Microbiological success/clinical failure, n (%)	1 (13)
Microbiological failure/clinical success, n (%)	2 (25)
Microbiological failure/clinical failure, n (%)	0
Participant level	
Success	5 (63) (24 - 91)
Failure	3 (38) (9 - 76)

A participant was counted more than once under a uropathogen category if multiple qualifying uropathogens within that uropathogen category were isolated at baseline for the participant. Other gram-negative bacilli consisted of *Citrobacter koseri* (1) and *Klebsiella pneumoniae* (1).

Abbreviations: CI, confidence interval.

[a]Clopper-Pearson CI.

TABLE S9 Analysis populations

Population	Definition	No. of participants included	Displays
Safety	Participants who took at least 1 dose of gepotidacin	22	Safety analyses
PK	Participants in the safety population and had evaluable plasma, urine, or swab/tissue concentration data for gepotidacin	22	Analyses and characterization of PK concentrations
PK parameter	Participants in the PK population who received gepotidacin 1500 mg BID for whom valid and evaluable plasma or urine PK parameters were derived for gepotidacin	22	Assessment and characterization of PK parameters
ITT	Participants who were assigned to study treatment	22	Summarizing disposition, baseline and demographic characteristics, reduction in susceptibility to gepotidacin, clinical symptom score, outcome and response, and clinical cure
micro-ITT	Participants in the ITT population, received at least 1 dose of gepotidacin, and had a qualifying baseline uropathogen from a quantitative bacteriological culture of a pretreatment clean-catch midstream urine specimen	8	Summarizing bacteriology results, susceptibility and MIC results, microbiological outcome and response, and PK/PD assessment
PKPD	Participants in both the PK parameter population and the micro-ITT population	8	None (data were summarized using the micro-ITT population)

Abbreviations: BID, twice daily; ITT, Intent-to-Treat; micro, microbiological; PD, pharmacodynamic; PK, pharmacokinetic.
